# Allosteric Modulation of the CB1 Cannabinoid Receptor by Cannabidiol—A Molecular Modeling Study of the N-Terminal Domain and the Allosteric-Orthosteric Coupling

**DOI:** 10.3390/molecules26092456

**Published:** 2021-04-23

**Authors:** Jakub Jakowiecki, Renata Abel, Urszula Orzeł, Paweł Pasznik, Robert Preissner, Sławomir Filipek

**Affiliations:** 1 Biological and Chemical Research Centre, Faculty of Chemistry, University of Warsaw, 02093 Warsaw, Poland; u.orzel@student.uw.edu.pl (U.O.); p.pasznik@gmail.com (P.P.); 2 Structural Bioinformatics Group, Institute for Physiology, Charité–University Medicine Berlin, 10115 Berlin, Germany; renata.abel@charite.de (R.A.); robert.preissner@charite.de (R.P.); 3 Department of Pharmaceutical Biology and Botany, Wroclaw Medical University, 50556 Wroclaw, Poland

**Keywords:** CB1, cannabinoid receptor, GPCRs, allosteric modulation, CBD, THC, interaction fingerprints, molecular dynamics, REST2

## Abstract

The CB_1_ cannabinoid receptor (CB_1_R) contains one of the longest N termini among class A G protein-coupled receptors. Mutagenesis studies suggest that the allosteric binding site of cannabidiol (CBD) involves residues from the N terminal domain. In order to study the allosteric binding of CBD to CB_1_R we modeled the whole N-terminus of this receptor using the replica exchange molecular dynamics with solute tempering (REST2) approach. Then, the obtained structures of CB_1_R with the N terminus were used for ligand docking. A natural cannabinoid receptor agonist, Δ^9^-THC, was docked to the orthosteric site and a negative allosteric modulator, CBD, to the allosteric site positioned between extracellular ends of helices TM1 and TM2. The molecular dynamics simulations were then performed for CB_1_R with ligands: (i) CBD together with THC, and (ii) THC-only. Analyses of the differences in the residue-residue interaction patterns between those two cases allowed us to elucidate the allosteric network responsible for the modulation of the CB_1_R by CBD. In addition, we identified the changes in the orthosteric binding mode of Δ^9^-THC, as well as the changes in its binding energy, caused by the CBD allosteric binding. We have also found that the presence of a complete N-terminal domain is essential for a stable binding of CBD in the allosteric site of CB_1_R as well as for the allosteric-orthosteric coupling mechanism.

## 1. Introduction

The cannabinoid CB_1_ receptor is the most abundant G protein-coupled receptor (GPCR) in the human central nervous system [1] and, together with CB_2_ receptor, is a part of the endocannabinoid system. Cannabinoid receptors participate in many biological processes, including mood regulation, appetite and lipid metabolism, cognitive functions, nociception, as well as cell growth and proliferation [2]. In particular, CB_1_R is a very attractive pharmacological target for treatment of many disorders [3], such as: multiple sclerosis, Parkinson’s disease, Huntington’s disease, epilepsy, pain, appetite deregulation and obesity [4,5]. Before the discovery of the allosteric binding site(s) in CB_1_R, designing new ligands for this receptor was solely based on the orthosteric binding site [6]. A long list of CB_1_R ligands including endocannabinoids, phytocannabinoids, and synthetic compounds have been discovered and developed over the last 20 years. In 2005, it was discovered that the CB_1_ receptor contains allosteric binding site(s) that can be targeted by small molecule ligands [7,8].

### 1.1. Advantages of Allosteric over Orthosteric Ligands of CB_1_R

**Figure 1:**
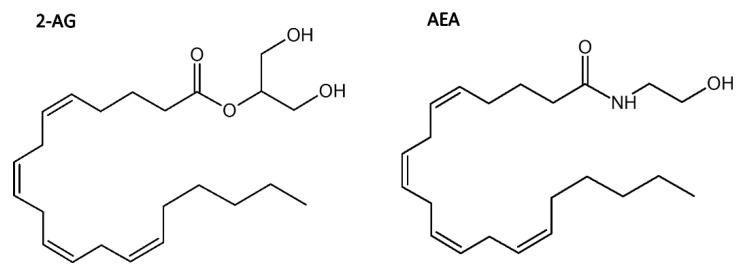
Chemical structures of endocannabinoids: 2‑arachidonylglycerol (2‑AG) and anandamide (AEA).

Considering the psychomimetic effects commonly associated with CB_1_R agonists and depressant effects associated with CB_1_ antagonists/inverse agonists, allosteric modulation may offer an alternative approach to attain potential therapeutic benefits while avoiding the unwanted side effects of orthosteric ligands [7]. Allosteric modulators do not possess intrinsic efficacy, but instead they enhance or diminish the receptor’s response to orthosteric ligands. In the absence of any exogenous CB_1_R orthosteric ligands the maximum effect of an allosteric modulator is determined by the level of endocannabinoids, such as anandamide (AEA) and 2‑arachidonylglycerol (2‑AG) (Figure 1) [9,10]. While the exogenous orthosteric ligands may produce adverse effects through long term over-activation or down-regulation of a receptor [9,10], the allosteric modulators of CB_1_R may not cause those undesirable effects since their efficacy depends on the presence of orthosteric ligands. Endogenous orthosteric ligands are released on demand and removed by enzymatic degradation (i.e., processed by fatty acid amide hydrolase (FAAH) [11]) or cellular reuptake when no longer needed. Therefore, the allosteric modulators allow for a tempering of cannabinoid receptor signalling without desensitization, tolerance or dependence [12]. In addition, the GPCR allosteric binding sites are often less conserved than orthosteric sites, thus the allosteric ligands have potentially greater specificity for particular receptor types and subtypes [7].

The allosteric ligands can be classified based on their effects on receptor signalling. There are positive allosteric modulators (PAMs), which enhance the orthosteric agonists signalling efficacy (independently of their effect on the biding affinity of the orthosteric ligand, which can be either positive, negative or null), and the negative allosteric modulators (NAMs), which attenuate the orthosteric agonists signalling. Apart from the modulators there are also different kinds of allosteric ligands, such as allosteric agonists capable of enhancing receptor activity even in the absence of an orthosteric agonist, allosteric antagonists that can supress receptor activation induced by orthosteric agonists (in a non-competitive manner) and, finally, neutral allosteric ligands (NALs), which bind to the allosteric binding sites without affecting receptor activity and affinity [13,14,15]. The activity of an allosteric modulator depends on the orthosteric probe used. Both the change in orthosteric ligand binding affinity and the magnitude of the allosteric modulation effect will vary between different orthosteric ligands [10,16].

### 1.2. Cannabidiol—Its Properties and the Binding Site

Cannabidiol (CBD) is one of the major phytocannabinoids present in *Cannabis* plants. Either alone or in a mixture with Δ^9^-tetrahydrocannabinol (Δ^9^-THC) it is being intensively studied as a safe and efficacious alternative for the treatment of various medical conditions such as cancer, multiple sclerosis, Parkinson’s (PD) and Alzheimer’s disease (AD). It has been recently approved in some countries for the treatment of some specific types of treatment-resistant childhood epilepsy [17]. The properties of CBD including their possible biological targets and docking studies were recently reviewed in [18].

Recently, it has been also proposed that CBD can have potential usage as a support drug for the treatment of COVID-19 disease [19,20,21,22]. There are several mechanisms (both CB_1_R/CB_2_R dependent and independent) that substantiate the potential benefits of CBD usage in anti-COVID-19 therapy. Firstly, the well-known anti-inflammatory and immunomodulatory activity of CBD that can inhibit an excessive immunological response leading to a cytokine storm, which is a direct cause of severe lung injury observed in many COVID‑19 patients [23]. It has been shown that CBD can ameliorate the symptoms of acute respiratory distress syndrome in mice through the upregulation of apelin [21] and studies on humans have shown a reduction of some pro-inflammatory monocytes production and of CD4 and CD8 T-cells activation frequencies in response to cannabinoids [20,24]. Secondly, CBD can possibly attenuate the crucial routes of SARS-CoV2 infection in humans since cannabis extract with a high CBD content was able to down-regulate the expression of two receptors needed for SARS-CoV2 entry into the cell: the angiotensin-converting enzyme 2 (ACE2) and the transmembrane serine protease 2 (TMPRSS2) [19,25]. Thirdly, CBD is a PPARγ (peroxisome proliferator-activated receptor-γ) agonist so it can display a direct antiviral activity. In addition, PPARγ agonists regulate fibroblast/myofibroblast activation and thus they can inhibit the development of pulmonary fibrosis [19]. Despite the fact that some of those reports sound very promising more data are needed to justify the use of CBD in the treatment of COVID-19.

**Figure 2:**
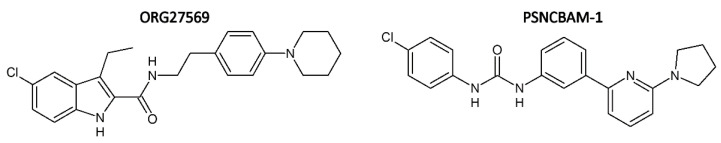
Chemical structures of CB_1_R allosteric ligands ORG27569 and PSNCBAM-1.

Although CBD is an isomer of THC (Figure 1) it has no psychoactive properties. In fact, it even suppresses some of the undesired side effects induced by Δ^9^-THC such as intoxication, sedation and tachycardia while attenuating its medical effects such as analgesic, anti-emetic, and anti-carcinogenic effects [26]. Together with terpenoids and other phytocannabinoids present in cannabis CBD contributes to the synergistic entourage effect [27]. CBD binds to a distinct binding site than the orthosteric ligands since it antagonises CB_1_R orthosteric agonists at concentrations far below its reported affinity [28]. The NAM activity of CBD was also demonstrated by Lapraire et al. [10] who showed that CBD reduces the potency and efficacy of Δ^9^-THC and 2-AG in a non‑competitive manner. Their results also indicate that the CBD allosteric binding site is located within the CB_1_R N-terminal domain and is particularly close to residues C98 and C107, which form a disulfide bond. They performed mutagenesis and BRET studies demonstrating that the C98A mutation as well as the C107A mutation led to a 50% loss of CBD NAM activity, versus both agonists, Δ^9^‑THC and 2-AG, when compared to WT CB_1_R. Interestingly, other mutations at those residues, C98S and C107S, caused no change in CBD NAM activity [10], which suggests that other interactions can substitute for the disulfide bridge and stabilize the local structure of CB_1_R. Presence of the Cys98-Cys107 bond also affects the allosteric activity of ORG27569 and PSNCBAM-1 (Figure 2) [29] and it is very likely that CBD binds to the same allosteric site as those two compounds. However, in contrast to ORG27569 and PSNCBAM-1, for which the ligand cooperativity is positive for agonists and negative for antagonists and inverse agonists [8,30], CBD displayed a negative cooperativity for all tested ligands [10].

### 1.3. The Importance of N-Terminus for Allosteric and Orthosteric Binding

**Figure 3:**
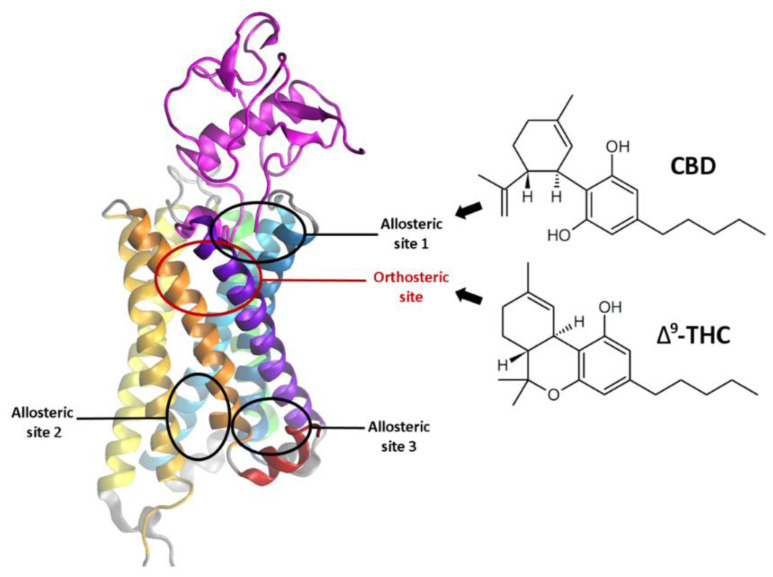
CB1 cannabinoid receptor with modelled N-terminus and the locations of orthosteric and allosteric binding sites. Cannabidiol (CBD)—an allosteric ligand, and Δ9-tetrahydrocannabinol (Δ^9^-THC)—an orthosteric ligand of CB1R. According to Lapraire et al. and Sabatucci et al. CBD very likely binds to the allosteric site 1 [10,31].

We have concluded that the role of the CB_1_R N-terminal domain in both allosteric and orthosteric ligand binding was significantly underestimated during recent years, and for this reason we decided to determine its structure as a whole, dedicating a special effort to modeling of the membrane-proximal region (MPR) and the allosteric binding site of CBD. The main inspiration for our project was the experimental work of Lapraire et al. on CBD binding [10]. In our research we have focused on exploring the CB_1_ receptor allosteric binding site (allosteric site 1) located between the extracellular ends of helices I and II (colored in blue and cyan in Figure 3, respectively). The three allosteric sites (Figure 1) were proposed by Sabatucci et al. [31] who found that CBD very likely binds to the allosteric site 1.

Sabatucci et al. performed docking studies in order to map the allosteric binding sites of CB_1_R [31]. However, as they point out themselves, for docking they used the crystal structures 5TGZ and 5U09, which lack most of the N-terminal residues, including C98, which has been shown to have an impact on allosteric modulation of the CB_1_ receptor [10,29]. Besides, they did not perform any molecular dynamics simulations that would confirm the stability of CBD binding in the allosteric site 1. A study on CBD allosteric binding that employed MD simulations has been done by Chung et al. [32]. They have reported an outward rotation of TM1 and TM2 upon CBD binding to the allosteric site 1, resulting in the expansion of the orthosteric binding site. They also revealed that CBD hydroxyl group formed interactions with D104 (which is in agreement with our results) while its alkyl chain formed hydrophobic contacts with F102, I105, I267 and F268. However, it should be pointed out that they used very short MD simulations, 25–50 ns, which were very likely insufficient for verification of CBD binding mode stability. Besides, those studies relied on crystal/cryo-EM structures supplemented with a small, proximal part of N-terminus only, without considering the influence of the complete N‑terminal domain on the allosteric binding.

**Figure 4:**
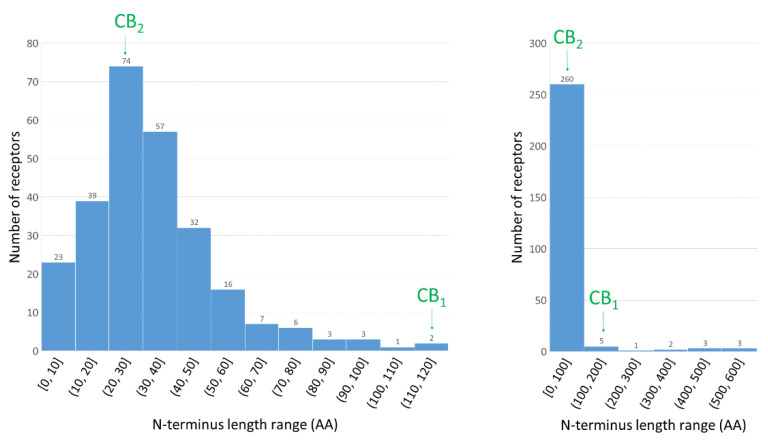
Distribution of N-terminus lengths within class A of GPCRs. On left the histogram with 10 amino acid interval; on right the histogram for all class A GPCR, without odorant receptors, with 100 amino acid interval. Number of receptors in each range is specified above each bar. The ranges are given in parenthesis which are open from left side. Based on GPCRdb data [33].

There is a large difference between lengths of N-termini of CB_1_ (111 residues) and CB_2_ (28 residues) receptors. We have done a comparison of N-termini of class A GPCRs excluding odorant receptors (Figure 4). The CB_1_R N-terminus is one of 14 longest N-termini of those receptors while for CB_2_R it has the most typical length. Because of this difference the allosteric-orthosteric coupling in both cannabinoid receptors is probably much different.

In our studies we have modeled the structure of the whole N-terminal domain of CB_1_R, docked CBD and THC to their binding sites, and run a set of long molecular dynamics (MD) simulations, 10 × 1 µs, to obtain statistically valid results. All the above simulations started from the same structure of CB_1_R with a complete N-terminus and THC bound to the orthosteric site. CBD was bound to the allosteric site 1 in five simulations, while in the other five simulations the allosteric site was empty. Analysis of the residue contact network and, especially, the differences in the interaction patterns between those two sets of trajectories, revealed interactions that are crucial for the allosteric-orthosteric coupling, called here the ‘allosteric network’. We also investigated the relevance of CB_1_R N-terminus for CBD allosteric binding by gradually truncating the N-terminus starting from its first residue and analyzing the changes in its stability and in the ligand binding energy. All performed MD simulations (including REST2 simulations) took over 150 μs (Table 1).

## 2. Results and Discussion

### 2.1. Construction of the N-Terminus of CB_1_R

**Figure 5:**
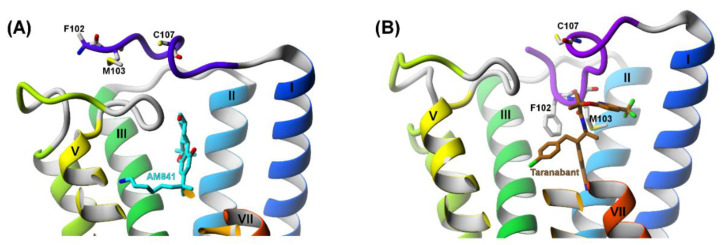
The influence of C98–C107 disulfide bond on the orthosteric binding of CB_1_R. (**A**) PDB id:5XR8 structure with no disulfide bond; (**B**) PDB id:5U09 structure with disulfide bond present. In 5XR8 structure the residues F102 and M103 are outside of the orthosteric binding site; the ligand’s position is shallower and there is much more space available in the orthosteric binding site so the ligand has more freedom and is in contact with many water molecules. In 5U09 structure the residues F102 and M103 are inside the orthosteric binding site; the ligand is positioned deeper, surrounded by hydrophobic residues and the binding is tighter.

The so called membrane-proximal region (MPR) of cannabinoid receptor (∼residues 90−110) has been proved to be crucial not only for the allosteric ligands binding, but also for binding of the orthosteric ligands [29]. According to Laprairie et al. [10] the 96–112 fragment (stabilized by the C98–C107 disulfide bond) is essential for the NAM activity of CBD. Therefore, we started with modeling of this small fragment. The task was made easier by the fact that the residues 100–112 were already present in the crystal structure of CB_1_R (PDB ID: 5U09 [34]), so only the 4 residues (96–99) were added. We chose this particular crystal structure because it had the longest N‑terminal part from all available experimental structures of that receptor. Also, it is very likely that this structure contains the disulfide bridge C98–C107 since the first residue occurring in the crystallized protein is G89, although the visible part of the structure starts from residue E100. Therefore, we assume that the geometry of the 100–112 fragment is the most correct among all available CB_1_R experimental structures and, hence, both the orthosteric and the allosteric binding sites are the most accurate and most resembling those of the wild-type receptor (Figure 5A,B). Choosing a correct initial structure at this stage is essential since the completely different residues constitute the particular binding sites in different crystal structures.

Different conformations of the binding site in the crystal structures of CB_1_R are not only a result of different orthosteric ligands bound but, most importantly, they ensue from the presence or absence of the MPR and, particularly, of the C98–C107 disulfide bond (Figure 5B). Such claim is in agreement with experimental results showing that mutations within MPR weaken the binding of CP55,940 to the orthosteric binding site [35] while truncation of the whole N‑terminus at position 113 abolishes the binding completely. However, if only MPR is retained, the binding of the orthosteric ligands is maintained [29].

**Figure 6:**
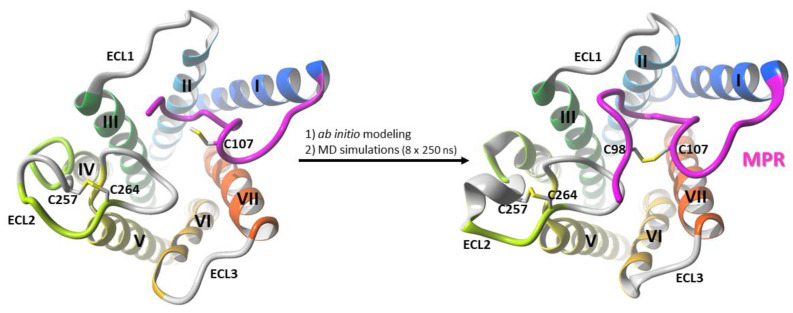
The modelling process of MPR of the CB_1_R N-terminal domain. Residues 96–99 were added to the CB_1_R crystal structure (PDB ID: 5U09) and the C98–C107 disulfide bond was created. Regions 96–100 and 107–108 were then sampled in Rosetta. Eight structures of CB_1_R with MPR obtained from Rosetta were placed in POPC bilayer and simulated for 250 ns.

We first elucidated the structure of MPR of CB_1_R (Figure 6)—see Section 3.1 of Methods for details. The structure of CB_1_R with MPR was used in our previous work [36] for investigating the entry pathway into the orthosteric binding site of the CB_1_ cannabinoid receptor. Next, the remaining residues 1–95 of the N-terminal domain were added according to the sequence of a wild-type human CB_1_R and the conformational space of the newly added part was extensively explored using the Replica Exchange Molecular Dynamics with Solute Tempering [37] simulations. Details of this procedure are specified in Section 3.2 of Methods.

### 2.2. Structural Features of the CB_1_R N-Terminal Domain

**Figure 7:**
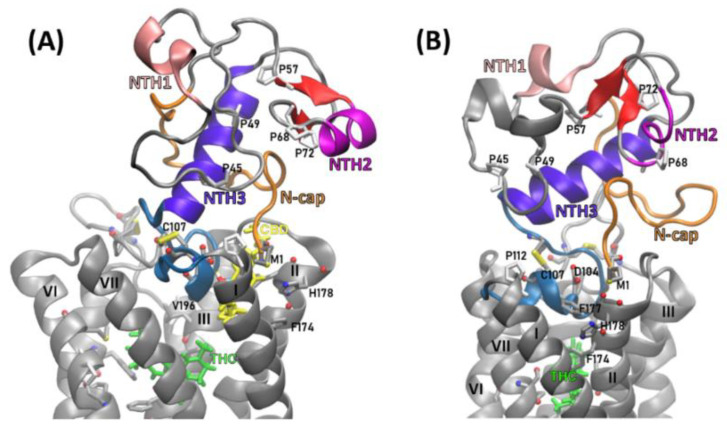
CB_1_ receptor N-terminal domain conformation depends on CBD presence in the allosteric site. (**A**) CB_1_R with CBD and THC bound, (**B**) CB_1_R with THC bound only; cyan—MPR, violet—N‑terminal helix 3 (NTH3, residues 80–94), red—a parallel β-sheet (residues 58–60 and 72–74), magenta—N‑terminal helix 2 (NTH2, residues 61–69), salmon—N‑terminal helix 1 (NTH1, residues 30–35), orange—N‑terminal cap (residues 1–20). Proline residues are shown as white sticks.

The N-terminal helix 3 (NTH3, residues 80–94) is present in many structures obtained from REST2 simulations and, once it forms, it is very stable (Figure 7A).

**Figure 8:**
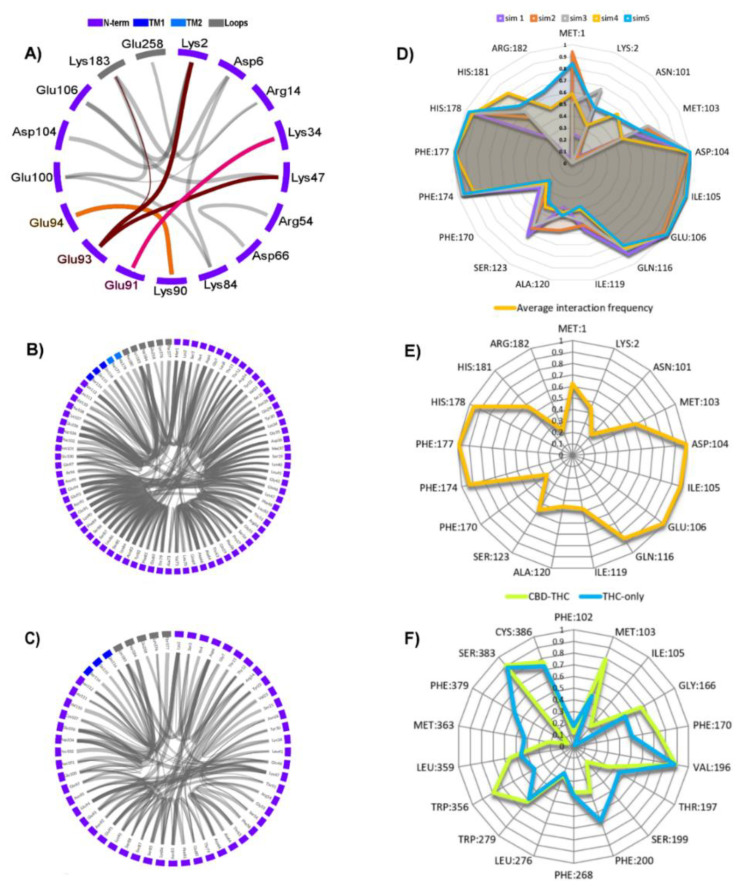
(**A**–**C**) Flare plots of crucial interactions responsible for stabilizing the N-terminal domain in THC-only complex). Line thickness is proportional to the frequency of the interaction; (**A**) Ionic interactions; highlighted are the interactions of E91 (pink), E93 (dark red) and E94 (orange), (**B**) Total hydrogen bonds of N-terminus; (**C**) The hydrogen bonds of N-terminus excluding backbone-backbone H-bonds; (**D**,**E**) Ligand-receptor dynamic interaction fingerprints in THC-CBD complex; (**D**) frequency fingerprints of interactions of residues with CBD for each simulation separately; (**E**) the average interaction frequencies of fingerprints from panel D; (**F**) the average frequency fingerprints of interactions of residues with THC for simulations with THC-CBD (green) and with THC-only (blue) bound to the CB_1_R.

**Figure 9:**
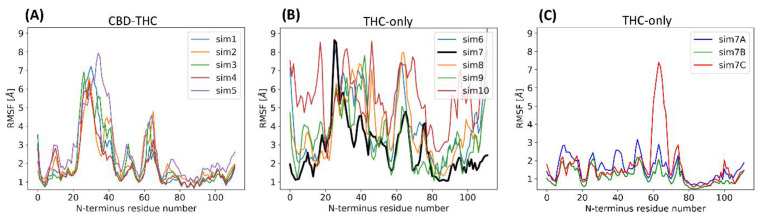
RMSF plots of CB_1_R N-terminus (residues 1–113) measured during 1 µs long MD simulations starting from the same receptor structure (Figure 4A) bound with (**A**) CBD and THC; (**B**) THC-only; (**C**) for simulations from N-terminus stability corroboration (Table 1). The last 1 µs from 3 µs MD simulations were taken for RMSF calculations.

Very likely this helix is a scaffold on which the rest of the N-terminus is arranged. The three glutamates, E91, E93, and E94, located in this helix, form ionic interactions with other parts of N-terminus (Figure 8A) constituting a hub essential for the N-terminal domain stability. For example, the 1–20 residue fragment, called by us the N-terminal cap (or simply ‘N-cap’) and located close to TM1 and TM2 extracellular ends, is relatively stable due to a salt‑bridge K2-E93 (and also K2-D104 as well as K2-E106) (see the interactions on Figure 8A) that supports the conformation and position of this fragment. The residues M1 and K2 are within the allosteric site 1, constituting a barrier separating that site from the extracellular milieu. We have also observed a short parallel β-sheet forming repeatedly between the 58–60 and 72–74 strands while a very short helix formed occasionally within the 60–72 loop. The N‑terminus contains five prolines: P45, P49, P57, P68 and P72, stacked together within a fragment of only 30 residues long. The stability of particular regions of N‑terminus were estimated by RMSF calculations (Figure 9A,B) based on ten 1 µs long MD simulations for CB_1_R complexes with and without CBD. The additional six MD simulations 3 µs long performed for corroboration of N-terminus stability gave the structure of increased stability for the CB_1_R complex with THC and without CBD (Figure 7B and Figure 9C) where two additional short helices were formed but helix NTH2 was dissolved in one of three simulations.

As we had anticipated, the five prolines-containing fragment disrupts a formation of the larger secondary structure elements in that region and only transient structures can be formed. The whole N-terminus is stabilized by internal salt bridges (Figure 8A) and a large number of internal hydrogen bonds but also forms a few interactions with the extracellular ends of TM1, TM2, and with the extracellular loops (Figure 8B,C).

### 2.3. The Orthosteric and Allosteric Ligand Binding

**Figure 10:**
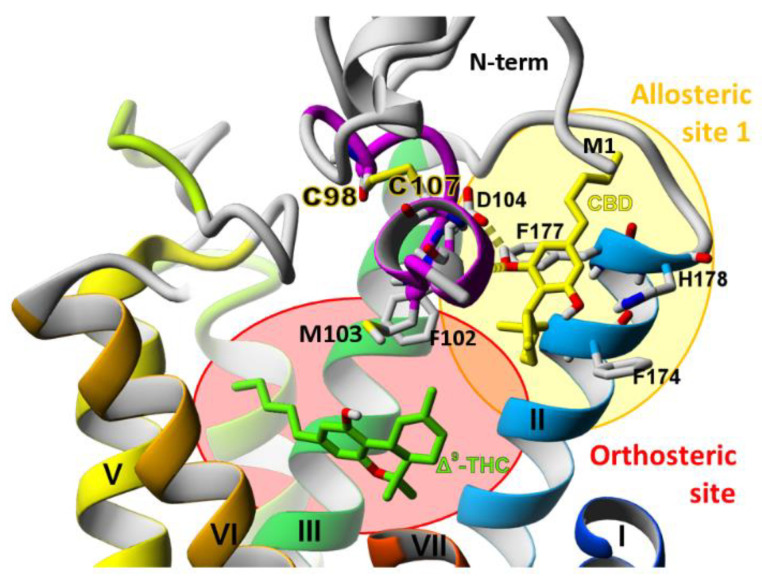
CBD and THC binding to CB_1_R. CBD is bound to the allosteric site 1 while Δ^9^-THC is bound to the orthosteric site. The allosteric site is located very close to the orthosteric binding site and they partially overlap one another. The MPR (purple) constitutes a barrier between the orthosteric and the allosteric binding sites. CBD bound to the allosteric binding site forms a hydrogen bond with D104 and π-π interactions with F177.

The analysis of ligand-receptor interaction patterns in the complex of CB_1_R with CBD and Δ^9^‑THC (Figure 10) during MD simulations demonstrates that the pose of CBD is very stable when it is bound to the allosteric site 1 of a wild-type CB_1_ cannabinoid receptor with a whole N-terminus which is indicated by existence of several ligand-receptor interactions with a frequency close to 1.0 for the residues D104, I105, E106 from MPR, Q116^1.32^ from helix I, and residues F174^2.61^, F177^2.64^, H178^2.65^ from helix II (Figure 8D,E). The important contribution to CBD binding comes also from the first residue of N-terminus, M1, which side chain partially protects CBD from the water environment.

The binding mode of THC in the orthosteric site is influenced by the presence of CBD in the allosteric site 1. The residues for which the frequencies of interactions with THC are not changed are V196^3.32^, T197^3.33^ of helix III, L276^5.40^, W279^5.43^ of helix V, L359^6.51^ of helix VI, and S383^7.39^, C386^7.42^ of helix VII (Figure 8F). CBD changes the average frequencies of interactions between THC and the receptor in such a way that some of them are increased and some decreased so the average binding pose of THC in the binding site is modified. The residues with increased frequencies are M103 of MPR, G166^2.53^, F170^2.57^ of helix II, and W356^6.48^ of helix VI. The residues with decreased frequencies are S199^3.35^, F200^3.36^ of helix III, and M363^6.55^ of helix VI (Figure 8F). The residue W356^6.48^ is a central residue of a transmission switch which is one of major molecular switches responsible for activation of GPCRs [38]. Molecular dynamics simulations for many GPCRs also suggest that the transmission switch is the first switch to be affected by the agonist binding [39,40]. Therefore, a modification of frequency of interactions between THC and the residues of the transmission switch (particularly F200^3.36^ and W356^6.48^ which are in tight proximity in CB_1_R) by CBD presence in the allosteric site most likely corresponds to the negative allosteric modulator properties of CBD.

We have also compared the calculated ligand binding energies. The average THC-binding energy is lower (more negative, which means a more stable complex) by around 7.5% in THC-only simulations (−53.9 kcal/mol) than in CBD-THC simulations (−50.1 kcal/mol). Such observation is in agreement with experimental results indicating that allosteric modulation of CB_1_R by CBD decreases the binding affinity of THC and of the other orthosteric ligands [10].

In this paper we use for residues located in transmembrane helices the additional numbers in superscript which is the Ballesteros–Weinstein numbering [41] (extension of this scheme was proposed by Gloriam group [42]). This system was proposed for GPCRs to provide information about the relative positions of amino acids present in seven transmembrane helices. It contains two numbers separated by a dot: the first number indicates the number of the transmembrane helix (1–7) where the residue is located; the second number indicates a position of particular residue in relation to the most conserved residue in that helix, assigned number 50. Such numbers allow for direct comparison of residues among different GPCRs and especially in the most populated class A.

### 2.4. Stability of the N-Terminal Domain

The N-terminal domain of CB_1_ receptor was stable in all five CBD-THC simulations, however, it was significantly less stable in the THC-only simulations (Figure 9A,B) and underwent a rearrangement (Figure 9C). It should be noted that in the last stage of REST2 simulations we used a CBD bound CB_1_R and, therefore, we believe that the conformation of the N-terminal domain accommodated the allosteric ligand. Although initially the N-terminus was less stable in the THC-only simulations when we continued one of the simulations (sim7 in Figure 9B) the N‑terminus structure converged to more stable conformation also containing NTH3 (residues 78–92), β-sheet between 58–60 and 72–74, NTH1 (residues 29–35) and a very stable ‘N-cap’ (residues 1–20) (Figure 7B). Therefore, we concluded that N-terminal domain is not only essential for stable binding of the allosteric ligands in ‘allosteric site 1’ but is also involved in allosteric modulation of the orthosteric binding. This hypothesis is supported by the mutagenesis studies of CB_1_R [29]. When this conformation has been achieved, we confirmed its stability in three independent 3 µs long MD simulations (Figure 9C). In one of the three simulations (sim7C) the NTH2 helix together with the β-sheet (residues 58–74) were dissolved and therefore we observe high RMSF values for this region in simulation 7C.

### 2.5. The Effect of Truncation of the N-Terminus on the Energy of CBD Binding

**Figure 11:**
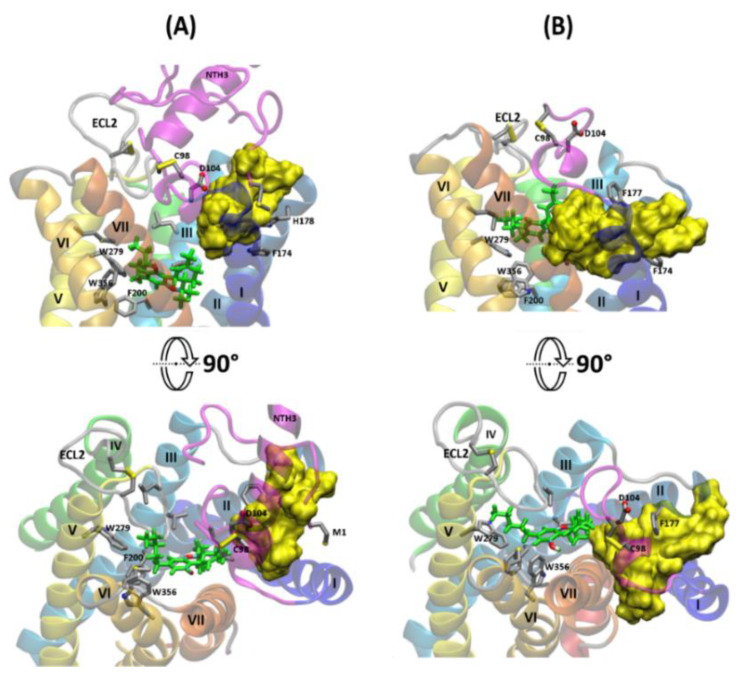
Comparison of CBD binding modes (yellow surface for all poses) obtained after 1 µs of MD simulations for (**A**) WT CB_1_R (with whole N‑terminus), five CBD poses superimposed; and for (**B**) CB_1_R with truncated N-terminus—four CBD poses (two for Δ-88 and two for Δ-98 CB_1_R). All poses were superimposed using receptor structure as a reference. Orthosteric agonist, THC, is shown in green.

The N-terminal domain of CB_1_R is essential for the stability of CBD in its allosteric binding site. In MD simulations of CBD-THC complex (5 × 1 µ and more) with a whole N‑terminal domain none of the ligands exited its binding site (the average RMSD for each ligand was ~2.5 Å). However, in all simulations of Δ-88 (deletion of 1–88 residues) and Δ‑98 (deletion of 1–98 residues) CB_1_R mutants (two simulations repeats for each mutant), the CBD binding mode was significantly distorted and the final pose was different for each of the four simulations (Figure 11). In two out of four cases (one simulation for Δ-88 and one simulation for Δ-98) CBD exited the allosteric site completely and within less than 500 ns it moved toward the orthosteric ligand binding channel between helices I and VII (this gate was described by us in our previous paper [36]) distorting the conformation of MPR significantly. In one of the two simulations for the Δ-8 (deletion of 1–8 residues) CB_1_R, CBD dissociated from the receptor completely after 350 ns and remained dissolved in the membrane for the rest of the simulation time.

**Figure 12:**
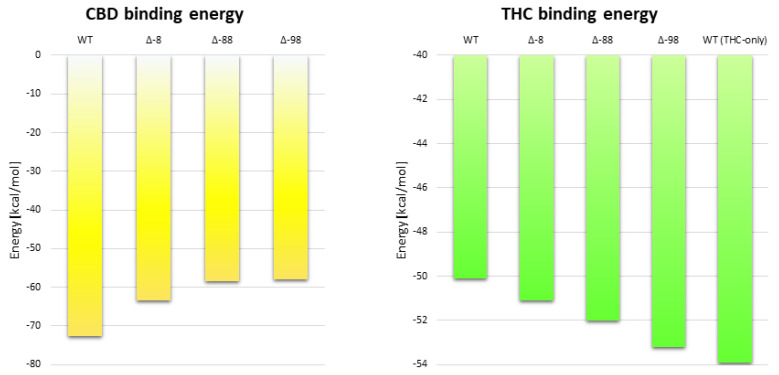
Comparison of ligand binding energies for different mutants of CB_1_R with truncated N‑terminus and a wild-type receptor for CBD-THC complex. The energies of CBD binding to the allosteric site (**left**) are shown in yellow while the energies of THC binding to the orthosteric site (**right**) are shown in green. All reported binding energies are the average values obtained from several (5 for WT, and 2 for mutants) 1 µs long MD simulations and they were performed for CBD-THC complex (except for the last rectangle). Δ-8 (deletion of 1–8 residues), Δ-88 (deletion of 1–88 residues) and Δ-98 (deletion of 1–98 residues).

The average RMSD of CBD was 12.5 Å for Δ-8 receptor, 5.7 Å for Δ-88, and 7.7 Å for the Δ−98 CB_1_R mutant and the average binding energies of CBD were −63.29, −58.49 and −57.83 kcal/mol, respectively, while for the wild‑type receptor the binding energy was −72.7 kcal/mol (Figure 12). The energies of CBD binding to mutants with a truncated N‑terminus (Δ-8, Δ-88, Δ-98) are significantly higher than for the wild-type receptor, suggesting that a whole N-terminus is required for proper CBD binding.

The average values of THC binding energy for Δ-8, Δ-88 and Δ-98 mutants (−51.1, −52.0 and −53.2 kcal/mol, respectively) are lower than those for THC bound to a wild-type receptor with CBD in the allosteric site (−50.1 kcal/mol) and higher than those recorded for THC bound to the wild-type receptor without CBD (−53.9 kcal/mol) (Figure 12). It suggests that even if CBD binds (at least to some extent) to CB_1_R with truncated N-terminus its impact on THC binding, and perhaps also its NAM activity, will be significantly reduced. Our results indicate that the larger fragment of the N-terminus is deleted the lower is the influence of CBD binding on the binding energy of THC (Figure 12).

### 2.6. The Dynamic Contact Networks

**Figure 13:**
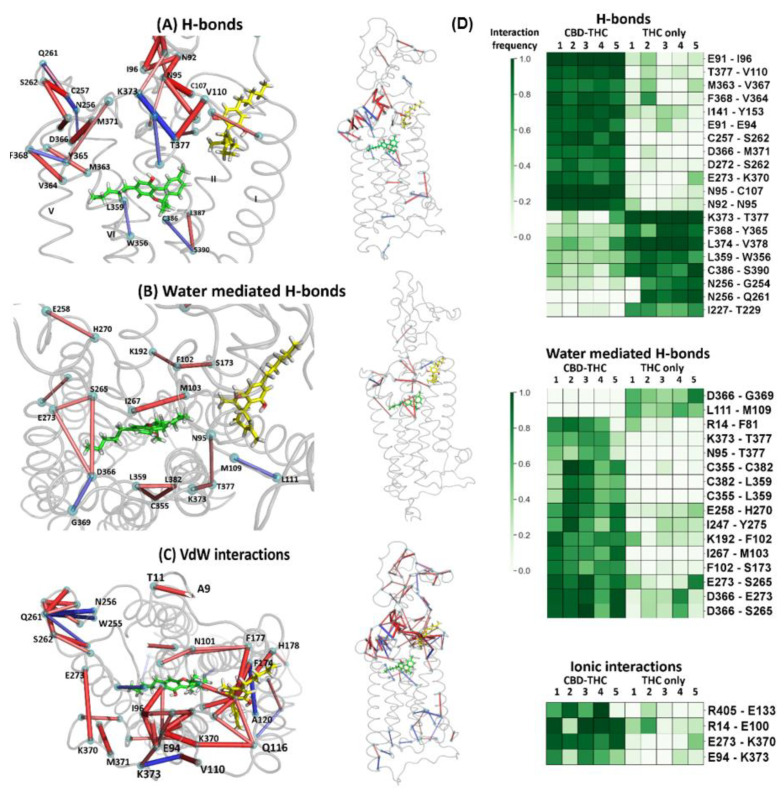
Differences in contact frequencies between CBD-THC and THC-only complexes; (**A**) Hydrogen bonds—both side views; (**B**) Water mediated hydrogen bonds—the extracellular and side view; (**C**) van der Waals interactions—the extracellular and side view. Red sticks represent contacts occurring more frequently for CBD-THC simulations, and blue ones are the contacts occurring more frequently in THC-only simulations. The thickness of a stick as well as a color intensity correspond to the average frequency difference between those two populations of trajectories; (**D**) The interaction frequency fingerprints for CBD-THC and THC-only complexes (5 simulations for each complex). Presented interactions are very likely participating in the allosteric-orthosteric communication network since their frequencies differ significantly between simulations with and without an allosteric ligand (CBD). Among all possible interactions only the hydrogen bonds, water mediated hydrogen bonds, and ionic interactions are shown.

The dynamic contact networks for CBD-THC and THC-only simulations are different. The most significant changes in frequencies of certain interactions in MD simulations are observed for hydrogen bonds, water mediated hydrogen bonds and van der Waals interactions networks (Figure 13). The interaction frequency and the interaction frequency difference (Δ*_freq_*) is defined in Section 3.5 of Methods.

**Figure 14:**
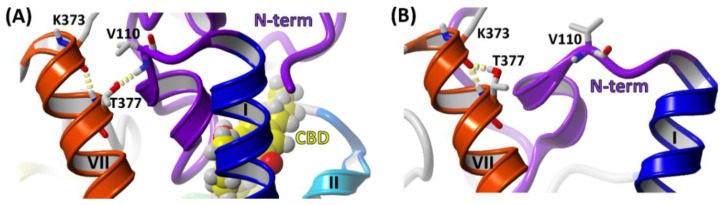
The molecular switch formed by residues V110^MPR^-T377^7.33^-K373^7.29^ as an important element of the allosteric modulation. (**A**) In CBD-THC simulations the switch exists predominantly in the configuration where a hydrogen bond V110^MPR^-T377^7.33^ (side chain-main chain) is formed; (**B**) in THC-only simulations the hydrogen bond of side chain of T377^7.33^ is much more often formed within the same helix VII to K373^7.29^ (side chain-main chain).

Comparison of the contact networks of CBD-THC and THC-only MD simulations has revealed that several residues from the N‑terminal domain (such as E91, E94, N95, I96, and C107) participate in the allosteric interaction network essential for the communication between the allosteric and orthosteric binding sites. Importance of disulfide bond C98–C107 for the allosteric-orthosteric coupling was strengthened since one of its residues, C107, formed a hydrogen bond with residue N95 in all MD simulations of CBD-THC complex while this bond was broken in all MD simulations of THC-only complex (Figure 13A,D). We have also found that the residues V110^MPR^, K373^7.29^ and T377^7.33^ constitute a switch that is involved in the allosteric modulation of CB_1_R by CBD. In the presence of CBD a hydrogen bond V110‑T377^7.33^ is predominantly formed (Figure 14A) while in absence of CBD a hydrogen bond K373^7.29^-T377^7.33^ is formed much more often (Figure 14B). For those interactions we observed one of the highest average frequency differences between CBD‑THC and THC‑only systems, 0.78 and −0.74, for V110‑T377^7.33^ and K373^7.29^-T377^7.33^, respectively (H-bonds in Figure 13A,D).

The residue M103^MPR^, which is located between the allosteric and orthosteric sites (Figure 10), interacts both with CBD in the allosteric site and with THC in the orthosteric site, and the frequencies of these interactions are high in CBD-THC complex: 0.61 for interaction with CBD (Figure 8E), and 0.80 for interaction with THC (Figure 8F). We conclude that M103^MPR^ could be relevant for the allosteric-orthosteric communication since the frequency of the M103-THC interaction is highly dependent on the presence or absence of CBD in the allosteric site, 0.80 in CBD-THC complex, and 0.46 in THC-only complex (Figure 8F). The residue I105^MPR^ is also located between the allosteric and orthosteric sites, however, the frequencies for I105-THC interaction are very low (Figure 8F), and therefore we cannot say if this residue participates in the allosteric-orthosteric coupling.

The four ionic interactions (Figure 13D), R405^H8^-E133^1.49^, R14^N-term^-E100^MPR^, E273^5.37^-K370^ECL3^, and E94^MPR^-K373^7.29^ were found significantly more often in the CBD-THC complex than in THC-only, with the average frequency difference (Δ*_freq_*): 0.46, 0.53, 0.54 and 0.75, respectively. Perhaps, the mutagenesis studies could elucidate the influence of the above residues on the allosteric-orthosteric coupling. The extracellular loop 2 (ECL2), and particularly the residues N256, C257, Q261, S262, also participate in the allosteric-orthosteric coupling mechanism especially that in some THC-only simulations we observed the interactions between N-terminal domain and ECL2 loop. Interestingly, not only the N-terminus residues but also some residues from cytoplasmic part of the receptor (Figure 7C) are involved in the allosteric modulation and can influence the cellular response.

## 3. Methods

### 3.1. Modeling of the Membrane-Proximal Region (MPR) of CB_1_R

The crystal structure of CB_1_R (PDB id: 5U09) was converted to the wild-type (T210A mutation reversed), the residues 96–99 were added using YASARA v.19.1.27 program [43] and the regions 96–100 and 107–108 were then sampled using the Rosetta loopmodel protocol [44] with a condition that the C98–C107 disulfide bond must be maintained. From the 500 models obtained eight were selected based on Rosetta total score. Those eight selected structures were placed into a hydrated POPC bilayer and 250 ns of a full atom MD simulation was performed in AMBER v.18 program [45] for each of them. In five out of eight simulations the 96–112 fragment converged to a similar stable structure and the one with the lowest nonbonded energy of MPR was selected for further studies.

### 3.2. Building and Elucidating the Structure of Whole N-Terminus

**Figure 15:**
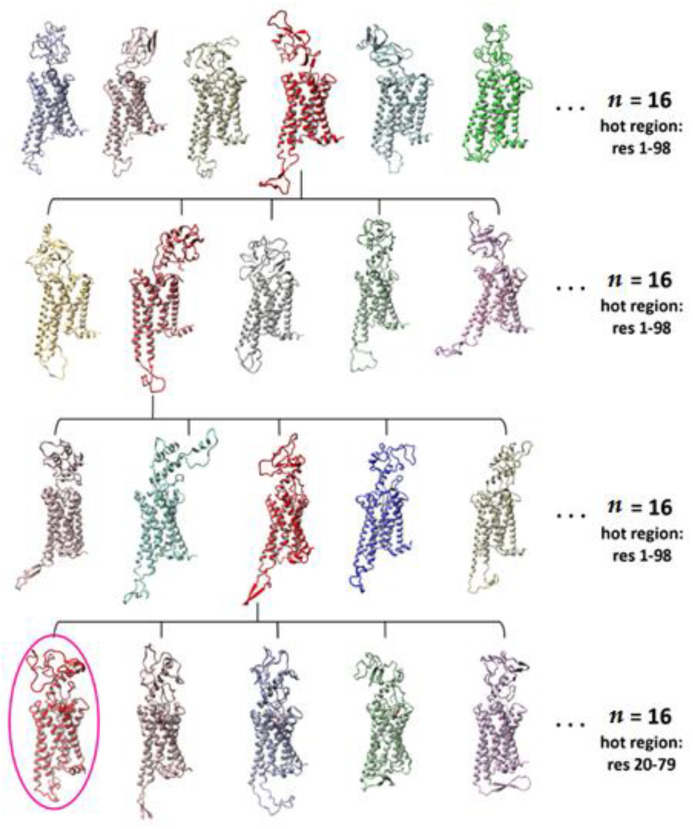
Sample structures of CB_1_ receptor with complete N-terminal domain at each selection step of the REST2 MD simulations. The span of hot region of N-terminal domain and number of replicas in each step are specified on the right.

The remaining 95 residues of the N-terminal domain (residues 1–95) were added from the sequence of a wild-type human CB_1_R obtained from the Uniprot database [46]. The BuildLoop command from YASARA [47] was used to build the initial model of N-terminus and its conformation was sampled using the Rosetta loopmodel protocol [44]. The selected structures from the clustered Rosetta solutions were used for exploration of the conformational space of the CB_1_R N‑terminal domain employing the Replica Exchange Molecular Dynamics with Solute Tempering (REST2 [37]) algorithm working within NAMD 2.13 platform [48] using CHARMM36m force field [49]. The all-atom REST2 simulations were performed for the whole CB_1_ cannabinoid receptor (residues 1–412) with the N-terminus (residues 1–98, unless specified otherwise) selected as a hot region (Figure 15).

The receptor was immersed in a hydrated 1-palmitoyl-2-oleoyl-sn-glycero-3‑phosphocholine (POPC) bilayer enriched by 20% (*n/n*) cholesterol. The whole system was generated in CHARMM-GUI webserver [50] which was used for system preparation and input files generation. The standard system contained ~70,000 atoms and ~130 lipid molecules (cholesterol + POPC) including Na^+^ and Cl^−^ counter ions in total ion concentration 0.15 M for system charge neutrality. The average dimensions of a periodic cell were 74 Å × 74 Å × 140 Å. The TIP3P water model, parametrized to use with CHARMM force fields, was employed. Each REST2 simulation was run in 16 replicas, all starting from the same initial structure. All simulations were performed using the 2 fs time step and all bond lengths to hydrogen atoms were constrained using SHAKE [51] algorithm allowing to use a longer time step than 1 fs. The actual temperature of the system was 300 K, while the interactions for the hot region were scaled for temperatures 300–600 K. Temperature exchange attempts were performed every 100 steps (200 fs). The electrostatic interactions cutoff was set to 12 Å with 10–12 Å switching function, while the cutoff for pairlist generation was set to 13.5 Å. Long-range electrostatic interactions were computed using the Particle Mesh Ewald [52] summation scheme with PMEGridSpacing parameter set to 1.0. In the above simulations the group pressure and the Langevin dynamics were used. The best structures of CB_1_R were elucidated from REST2 simulations based on their lowest nonbonded energies and low local RMSF values of the N-terminus during the simulations. For the last step of REST2 simulation the residues 20–79 instead of 1–98 were selected as the hot region due to their high RMSF values and CBD was docked to the allosteric site (Figure 15).

### 3.3. Ligand Docking

The most stable structure of CB_1_R with the whole N-terminus obtained from REST2 simulations and selected based on RMSF and energy analysis, was used for ligand docking. CBD was redocked to the allosteric site 1 [31] (positioned between the extracellular ends of TM1 and TM2, defined by residues: I105, E106, C107) after the last step of REST2 set of simulations, while Δ^9^‑THC was docked to the orthosteric binding site (defined by residues: I119, F174, L193, W356, F381, S383, M384) [34]. To perform docking of flexible ligands into protein binding sites we used Genetic Optimization for Ligand Docking (GOLD) v.5.7.3 [53]. Ligand poses were verified for stability by MD simulations. Only one stable pose was identified for CBD and it was stable for >1 μs of total MD simulation (in 5 simulation repeats). For each of the two ligand-receptor complexes (1st complex of CB_1_R with CBD and THC, and 2nd complex of CB_1_R with THC only) five independent all-atom and unbiased MD simulations were performed (2 × 5 × 1 μs).

### 3.4. All-Atom MD Simulations of CB_1_R Receptor with Ligands

Standard molecular dynamics simulations of the CB_1_ cannabinoid receptor with appropriate ligands immersed in a hydrated POPC bilayer (enriched by 20% (*n/n*) cholesterol), were performed in AMBER18 using CHARMM36m force field [49]. All systems were generated in CHARMM-GUI service [50] and the receptor orientation in the membrane was set according to the OPM database [54]. The standard system contained ~70 000 atoms, ~127 lipid molecules (cholesterol + POPC), 43 Na^+^ cations and 50 Cl^−^ anions (total ion concentration was 0.15 M). The average dimensions of a periodic cell were 74 Å × 74 Å × 140 Å. TIP3P water model parametrized to use with CHARMM force fields, was employed. Before each MD simulation the system was submitted to a restrained energy minimization (5000 cycles). The first 2500 minimization cycles were performed with a steepest-descent method and after that conjugate gradient was switched on. Next, a six‑step equilibration was performed (375 ps total) at a constant pressure and temperature (NPT ensemble; 310 K, 1 bar). During equilibration the restraints were released gradually, until the last step (last 100 ps of equilibration), in which no restraints were used. In the production simulations (as well as in the last three equilibration steps) all bond lengths to hydrogen atoms were constrained using SHAKE algorithm [51,55], which allowed us to use a longer time step (2 fs instead of 1 fs). Van der Waals and short-range electrostatic interactions cutoff =12 Å, with 10–12 Å switching function, were used. Long-range electrostatic interactions were computed using the Particle Mesh Ewald [52] summation scheme.

The MD simulations of 1 µs lengths each were performed for the CB1 receptor with the complete N-terminus (model obtained from REST2 simulations) and the following ligands bound: (1) five simulations for Δ^9^-THC in the orthosteric binding site and CBD in the allosteric binding site, and (2) five simulations for Δ^9^-THC in the orthosteric binding site and without the allosteric ligand.

### 3.5. Contact Network Analysis

The Pytraj tool (https://github.com/Amber-MD/pytraj (accessed on 6 March 2021)), a Python front-end of the popular cpptraj package [56], was used for trajectory processing. GetContacts software (https://getcontacts.github.io/ (accessed on 6 March 2021)) was used for analysis of the contact network within the receptor, and for calculation of the ligand-receptor and receptor-receptor interaction frequencies in each MD trajectory. The Flare plots (https://gpcrviz.github.io/flareplot/ (accessed on 6 March 2021)) were used to visualize the residue‑residue interactions. Based on the trajectory from the MD simulations using get_dynamic_contacts.py tool, we created a residue contact list for the MD simulation range 100 ns–1 µs. Since all MD simulations started from the same structure of CB_1_R selected using the REST2 simulations, by skipping the first 100 ns we hoped to highlight the differences between the contact networks of receptors bound with a different set of ligands. Using get_contact_frequencies.py module we calculated contact frequencies for all interactions in each trajectory. The interaction frequency (*Freq*) ranges between 0 and 1: *Freq* = 1.0 means that a particular interaction is present in every single trajectory frame, *Freq* = 0.5 means the interaction is found only in 50% of the frames, while *Freq* = 0 means the interaction does not occur in the analyzed trajectory. By comparing contact frequencies between two sets of trajectories (1st for CB_1_R with CBD and THC, and 2nd for CB_1_R with THC-only; 5 trajectories for each set of ligands) we constructed the interaction fingerprints. The cutoff for residue-residue interaction fingerprint was set to 0.5 (which means that a particular interaction is considered only if its frequency is ≥0.5 in at least one of the trajectories) and for ligand-protein interaction fingerprints the cutoff was set to 0.3.

Based on the analysis of numerical interaction fingerprints, we elucidated the interactions that are highly dependent on the presence or absence of CBD in the THC bound CB_1_ receptor. We performed a statistical analysis of the frequency distributions in two sets of trajectories and calculated the difference between average frequencies of a particular contact (Δ*_freq_*, described by Equation (1)) in order to identify those contacts for which the frequency differed significantly between CBD-THC and THC-only CB_1_ receptor complexes. The Student’s t-test was performed to verify whether the distributions of frequencies are different between two sets of trajectories comparing the average frequencies of each contact between those sets. If the frequency distributions are classified as different (*p* value < 0.05) and the absolute value of the average frequency difference between the two populations of trajectories is higher than 0.4 (|Δ*_freq_*| > 0.4) for a particular contact, then the contact is kept as a potentially meaningful for the allosteric‑orthosteric coupling mechanism. We visualized the average frequency differences for these meaningful interactions as red and blue sticks linking the interacting residues (Figure 13A–C).

(1) $$\Delta_{freq}=\bar{Freq_{\left(CBD+THC\right)}}-\bar{Freq_{\left(THC-only\right)}}$$

### 3.6. Mutational Study of N-Terminus on CBD Binding to the CB1 Receptor

In order to verify if the presence of the N-terminus is relevant for the allosteric binding of CBD to the CB_1_ receptor, we performed all-atom MD simulations of 1 µs lengths for CB_1_R with N-terminus truncated at various sites (Δ-8, Δ-88, Δ-98) bound to CBD (in the allosteric site 1) and THC (in the orthosteric site). Then, we compared the binding energies and the RMSD values of ligands in all CB_1_R mutants with those values for ligand-bound wild-type receptor.

## 4. Conclusions

This is the first reported attempt of model the whole N-terminal domain of CB_1_R concluded by obtaining a relatively stable structure of this domain maintained throughout multiple MD simulations. Although for some parts of the N-terminus the RMSF values are in range 7–8 Å it also contains very stable regions with RMSF in range 1–3 Å. Besides, it cannot be ruled out that some regions of the N‑terminus are flexible by nature and their conformation is highly sensitive to the allosteric ligand bound to allosteric site. This might explain why no experimental structure of the CB_1_R N‑terminal domain has been determined so far. We observed that the particular conformation of N-terminus obtained for CBD-THC-CB_1_R complex was stable in all five simulations but it underwent a rearrangement in all five simulations of the THC-only-CB_1_R complex. Continuation of one of these MD simulations (4 µs in total) led to a different stable conformation of the N-terminus of the THC-only-CB_1_R complex.

We found that CBD bound to the allosteric site is stable only when the whole N-terminus of CB_1_R is present. In the case of Δ-8, Δ-88 and Δ-98 mutants (deletion of 1–8 residues, 1–88 residues, and 1–98 residues, respectively), the binding energy of CBD is significantly higher, indicating a less stable complex, and the ligand dissociates from the binding site in 50% of cases. By analyzing of MD simulations of CBD-THC and THC-only complexes, and comparing the residue contact networks within those systems, we elucidated the major interactions responsible for the allosteric modulation of CB_1_R by CBD: (i) the N-terminus residues with a possible molecular switch involving residues V110^MPR^, K373^7.29^ and T377^7.33^ with alternating hydrogen bonds between them, and (ii) the residues from the extracellular loop 2 (ECL2).

The results of MD simulations indicate that the N-terminal domain of CB_1_R plays a critical role not only in the binding of CBD (and perhaps the other allosteric modulators) but also for the allosteric-orthosteric coupling mechanism. Better understanding of the role of the CB_1_R N‑terminal domain, including elucidation of the allosteric-orthosteric interaction network, is extremely important for understanding the allosteric modulation mechanism in CB_1_R and can be potentially useful in a design of novel allosteric modulators. All of the above findings on the role of N-terminal domain need the experimental confirmation but they are also useful on their own for molecular modelers and also for drug discovery and design purposes.

## Figures and Tables

**Table 1:** The MD simulations performed in this work.

MD Simulation Type	Average Length [μs]	Repeats	REST2 Replicas	Cumulative Length [μs]
CB_1_R with MPR	0.25	8	1	2
REST2 of CB_1_R with complete N-terminus	1.2	6	16	115.2
CB_1_R with CBD-THC (for contact map)	1	5	1	5
CB_1_R with THC-only (for contact map)	1	5	1	5
CB_1_R N-terminus stability corroboration (with and without CBD)	2 × 3	3	1	18
CB_1_R with truncated N-term (mutation studies)	3 × 1	2	1	6
**Total**				**151.2**

## Data Availability

The data supporting reported results are deposited at Faculty of Chemistry, University of Warsaw, and are available upon request.

## Sample Availability

The structures and trajectories of CBD-THC-CB_1_R and THC-only-CB_1_R complexes are available from the authors.

## Abbreviations

2-AG	2-arachidonylogycerol
AEA	N-arachidonoylethanolamine (anandamide)
CB_1_R	CB_1_ cannabinoid receptor
CBD	cannabidiol
COVID-19	coronavirus disease 19
GPCR	G protein coupled receptor
MD	molecular dynamics
MPR	membrane-proximal region
NAM	negative allosteric modulator
PAM	positive allosteric modulator
REST2	replica exchange. molecular dynamics with solute tempering
RMSD	root mean square deviation
RMSF	root mean square fluctuation
THC	(−)-trans-Δ^9^-tetrahydrocannabinol
